# Psychological morbidity among coal miners compared to other occupations in Appalachia

**DOI:** 10.1186/s12995-024-00439-0

**Published:** 2024-10-22

**Authors:** Paul D. Blanc, Laura Trupin, Edward H. Yelin, Patricia P. Katz

**Affiliations:** 1 Division of Occupational, Environmental and Climate Medicine, Department of Medicine, University of California San Francisco, San Francisco, Box 0834, CA USA; 2 Division of Rheumatology, Department of Medicine, University of California San Francisco, San Francisco, CA USA

**Keywords:** Miners, Coal worker’s pneumoconiosis, Depression, Anxiety, Post-traumatic stress disorder, Occupational trauma

## Abstract

**Objectives:**

Depressive symptoms, anxiety, and post-traumatic stress disorder (PTSD) are common morbidities among coal miners, but the risk of these morbidities has not been analyzed relative to other occupations taking into account relevant covariates.

**Methods:**

Using random digit dialing, we surveyed men aged 50 or over with a history of employment who resided in counties in Appalachia with high coal workers’ pneumoconiosis (CWP) mortality rates. We used the Primary Care Post-Traumatic Stress (PTSD) Screen and the Brief Trauma Questionnaire to query specific traumatic experiences. We used the Patient Health Questionnaire scale to assess depression symptoms and the Generalized Anxiety Disorder questionnaire to measure anxiety. Multivariable logistic regression analyzed associations between coal mining and depression, anxiety, and PTSD, adjusting for trauma, smoking and demographics.

**Results:**

Of 1,428 participants, 233 (16.3%) reported coal mining employment. Coal mining was associated with increased odds of depression (OR 1.6; 95% CI 1.1 to 2.4) and anxiety (OR 1.7; 95% CI 1.1 to 2.7). Among those with any trauma (*n* = 711), coal mining was not associated with increased risk of PTSD (OR 0.80; 95% CI 0.5 to 1.3]. Non-coal trauma was associated with three-fold increased odds of anxiety (OR 3.2; 95% CI 2.0 to 5.1); for coal trauma, anxiety was associated with six-fold increased odds (OR 6.0; 95% CI 2.9 to 12.4).

**Conclusions:**

Appalachian region coal miners carry an increased burden of depression and anxiety. This should be recognized by clinicians and at a population level, as worthy of individual and public health intervention.

## Background

Depressive symptoms, anxiety, and post-traumatic stress disorder (PTSD) comprise major sources of potential morbidity in working populations [1–3]. This is especially notable among U.S. coal miners, based on a 2021 study in Appalachia reporting that 37% of current and former miners surveyed from a black lung (coal workers’ pneumoconiosis [CWP]) clinic population reported symptoms consistent with a depression disorder; 40% provided responses consistent with clinically significant anxiety; and 26% screened positively for PTSD [4]. Mining is well recognized as a particularly dangerous occupation, marked by both increased risk of work-related physical trauma and occupational disease. Although those factors could account for an elevated prevalence of psychological morbidity, the inter-relationships among work-related trauma, depression, anxiety, and PTSD in this high-risk population have not been well elucidated [5]. Moreover, the role that living in an economically depressed region such as Appalachia may play raises the question of psychosocial morbidity across other occupations even without the same degree of work-related trauma and illness risk.

Addressing these open questions is important to fully assess the health needs not only of coal miners, but also of their families, communities, and other occupational groups. We sought to estimate the prevalence of PTSD, depression, and anxiety among current and former coal miners, analyzing whether coal mining is an independent risk factor for these morbidities in comparison with other occupations. To do so, we carried out a population-based survey of adult males in the Appalachia region, independent of health status or medical care, ascertaining symptoms and modeling occupational predictors of PTSD, depression, and anxiety.

## Methods

Data for this study derive from a population-based telephone survey of men aged 50 or over residing in counties in Appalachia (including parts of Kentucky, Ohio, Pennsylvania, Tennessee, Virginia, and West Virginia) with historically high mortality rates from CWP, based on data from the National Institute for Occupational Safety and Health (NIOSH) [6]. We limited eligibility to males aged 50 or over to conform to our previous population-based protocols that successfully recruited coal worker participants in sufficient study numbers [7, 8]. The survey sample included landline and cellphone listings. It was drawn from two prior random digit dial (RDD) surveys using the identical sampling frame along with newer listings in order to reach the desired sample size of 1,500. We limited eligibility for survey participation to English language speakers with a history of any past or current labor force participation and confirmed current residence in one of the targeted counties. The study was approved by the Institutional Review Board of the University of California San Francisco; all participants provided verbal consent to proceed with the interview.

### Study sample

**Fig. 1:**
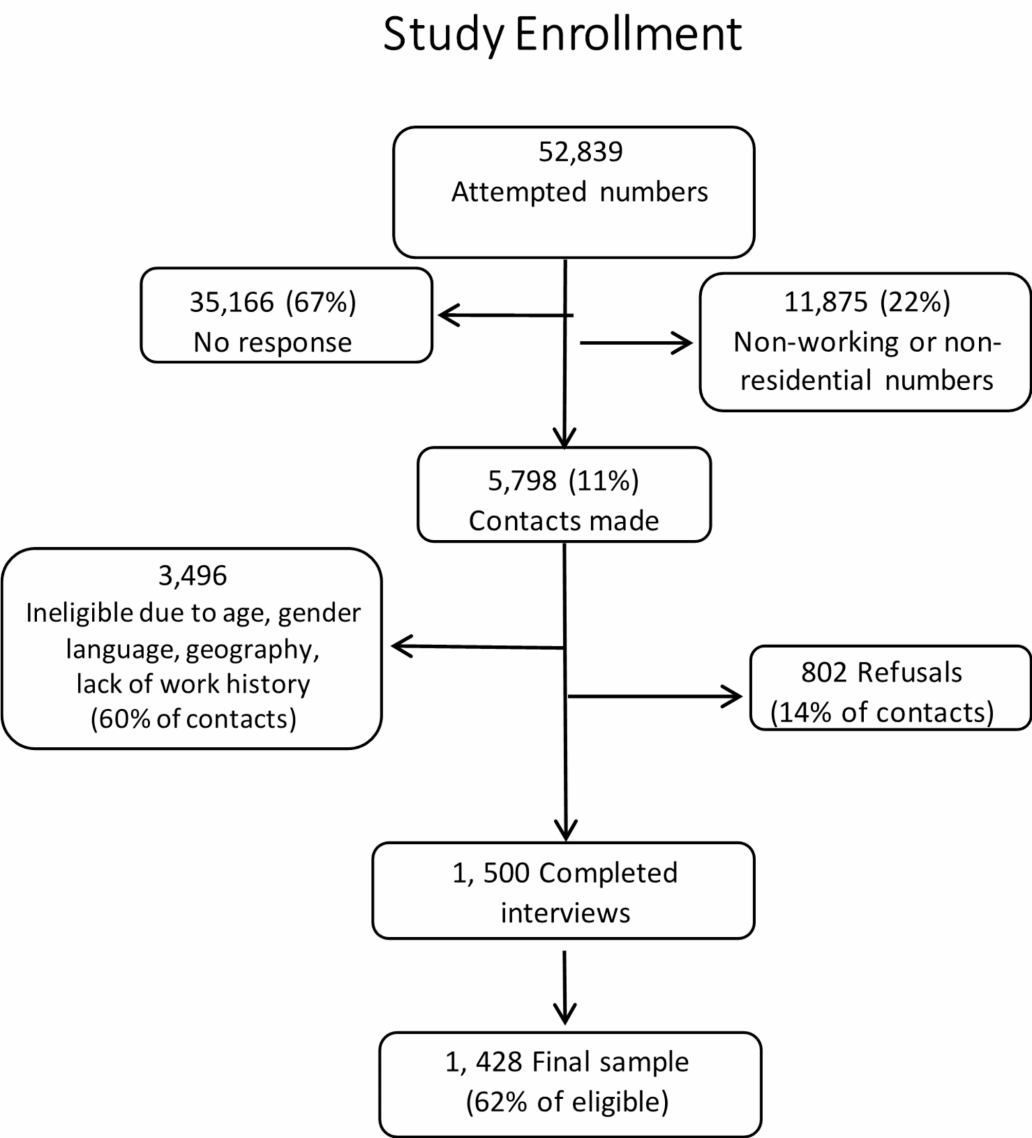
Participant study enrollment flow

From 52,839 call attempts, we made 5,798 contacts with potential participants (Fig. 1). There were 3,494 who were ineligible due to age, sex, language (non-English speakers), lack of employment history, or current residence outside the catchment area. There were 802 individuals who declined to participate after eligibility was established. A total of 1,500 responded to the survey, of whom 72 (5%) were dropped from the analysis due to missing values for one or more key variables, leaving a final sample of 1,428 (62% of eligible).

### Survey instrument

Study interviews averaged 15 min in administration length and contained items addressing employment, sociodemographics, self-assessed health status, smoking history and ethanol use. We also ascertained measures of trauma exposure, PTSD screening responses, and depression and anxiety questionnaire items. The survey ascertained duration and type of mining experience and queried whether the respondent had ever been given a diagnosis of black lung disease by a health care provider.

### Outcome and exposure measures

We used the 5-item Primary Care Post-traumatic Stress Screen (PC-PTSD55) to identify experiences of traumatic events and potential post-traumatic stress responses [9]. This screener has been used in many clinical settings as a tool to identify individuals at risk for PTSD. A score of 3 or higher, out of 5 total points, is considered indicative of PTSD. An introductory item provides examples of the traumatic events that includes “a serious accident or fire; a physical or sexual assault or abuse; an earthquake or flood; a war; seeing someone be killed or seriously injured; having a loved one die through homicide or suicide.” Only those who respond affirmatively to experiencing an event of this nature were asked the PTSD battery. In addition, to elicit more detailed information about trauma, the survey instrument included a modified version of the Brief Trauma Questionnaire, which queried specific traumatic experiences, such as war, automobile or work accidents, technological or environmental disasters, experience of physical violence, or childhood sexual abuse [10]. Where appropriate, follow-up questions determined if the events were related to their work as a coal miner for those with mining experience, and/or if they were related to work more generally. From these questions, we categorized respondents into four groups: those with no trauma reported, with only non-work-related trauma, with only non-coal mining work-related trauma, and those with coal mining related trauma. Respondents with no coal mining experience could only appear in the first three groups.

We used the eight-item Patient Health Questionnaire (PHQ-8) scale to estimate depressive symptoms and the seven-item Generalized Anxiety Disorder (GAD-7) questionnaire to measure anxiety [11–13]. Both measures have been extensively validated for use in public health surveys. For this analysis, we used established cut-points of 10 for both measures to define clinically meaningful levels of depression and anxiety symptoms, respectively.

The primary exposure variable for the current analysis was current or past work in coal mining. Coal mining exposure was further categorized as underground or surface mining. We also ascertained respondent report of receiving a diagnosis of CWP from a health care provider.

### Statistical analysis

We tabulated characteristics for the total sample and compared characteristics of respondents with and without a history of coal mining using chi-square analyses for categorical variables and t-tests for continuous variables. We compared traumatic experiences captured in the Brief Trauma Questionnaire among respondents with and without coal mining experience, using chi-square tests. Next, we examined the associations between coal mining and the three mental health outcomes: depression (PHQ-8 ≥ 10), anxiety (GAD-7 ≥ 10) and post-traumatic stress (PC-PTSD-5 ≥ 3). We estimated odds ratios and 95% confidence intervals from logistic regression models, with and without adjustment for trauma (work-related or non-work related), age, education, smoking (ever), and obesity (BMI ≥ 30 based on self-reported height and weight). Because PTSD is conditioned on experience of trauma, the analyses for that variable were limited to respondents who reported traumatic experiences in the screener. Turning to the four-level source of trauma rather than the dichotomous measure of coal mining experience, we examined the associations between source of trauma and the three outcomes, in logistic regression models with and without adjustment for age, education, smoking, and obesity. As a sensitivity analysis, we re-estimated the model for anxiety excluding participants who reported a diagnosis of CWP. Statistical analyses were carried out in SAS v9.4.

## Results

Table 1 shows the characteristics of the 1428 study participants included in the analysis by coal mining status. Of 1428, 233 (16.3%) reported coal mining employment. Age, race/ethnicity, and the proportion of ever smokers were similar between the miners and all others. Coal miners, however, had statistically significantly lower educational attainment and lower household incomes (*n* = 96 missing responses for income) compared to the non-coal miner study participants. Among the coal miners 48% self-reported fair or poor health compared to 28% of non-miners (*p* < 0.001). A greater proportion of miners were obese, but they were less likely to have high levels of alcohol consumption (Table 1).

**Table 1:** Study population characteristics by coal mining status

	All	Any Coal Mining Employment	No Coal Mining Employment	
	(n = 1428)	(n = 233)	(n = 1234)	*p*-value
Age, mean ± sd	66.4 ± 9.2	67.2 ± 8.3	66.3 ± 9.4	0.17
Race/ethnicity				0.99
White	1326 (94%)	211 (94%)	1115 (94%)	
Black	25 (2%)	4 (2%)	21 (2%)	
All other races (includes Hispanic ethnicity)	61 (4%)	10 (4%)	51 (4%)	
Education				< 0.001
HS or less	315 (22%)	83 (37%)	232 (19%)	
Some college, no 4 year degree	544 (38%)	94 (41%)	450 (37%)	
4 year college degree or more	569 (40%)	50 (22%)	519 (43%)	
Household income				< 0.001
< $40,000	383 (29%)	81 (38%)	302 (27%)	
$40,000–79,999	432 (32%)	74 (35%)	358 (32%)	
$80,000 or more	517 (39%)	56 (27%)	461 (41%)	
Self-reported fair or poor health	447 (31%)	109 (48%)	338 (28%)	< 0.001
Obesity (BMI ≥ 30)	659 (46%)	122 (54%)	537 (45%)	0.01
Ever smoker	752 (53%)	121 (53%)	631 (53%)	0.83
Alcohol consumption > 28 drinks/month	200 (14%)	21 (9%)	179 (15%)	0.02

Cells are n(%) unless indicated

Statistical tests: χ2 for categoric variables, Mantel-Haenszel χ2 test for ordinal, t-test for continuous (age)

Number of missing values: race/ethnicity (16), income (96), years of exposure (19)

Of the 233 coal miners, the median duration of mining employment was 12 years (interquartile range 3–29 years). The majority (59%) had engaged in some underground coal mining; the remainder had only worked in surface mining. One in four (56) reported having received a diagnosis of CWP. Only 12 (5%) of the respondents were still employed as coal miners.

The prevalence of past traumatic events, by type and severity, is shown in Table 2. Of 1428 participants, 50% (711) reported any past traumatic event consistent with the PTSD screening questionnaire (see Methods). Trauma was similar but statistically more frequent among those who ever worked in coal compared to all others (56% vs. 49%; *p* = 0.04). Work-related trauma was more frequent in the coal worker group, with both coal specific (21%) and non-coal work trauma (14%) contributing to the 35% prevalence. Trauma from any physical accident (including vehicular and occupational) was more common among the coal worker group, and this difference was particularly marked for work-related trauma (21% vs. 8%; *p* < 0.001). The coal miners more frequently had witnessed trauma (26% vs. 15%; *p* < 0.001); the majority (39 of 60) had witnessed the trauma specifically in coal mining.

**Table 2:** Traumatic events Associated with Work- and non-work-related events

	All	Any Coal Mining Employment	No Coal Mining Employment	
	(n = 1428)	(n = 227)	(n = 1201)	
	n (%)	n (%)	n (%)	p-value
Any Traumatic Event	711 (50%)	127 (56%)	584 (49%)	0.04
Work-Related Trauma				0.001
No traumatic event	717 (50%)	100 (44%)	617 (51%)	
Only non-work related event(s)	364 (25%)	48 (21%)	316 (26%)	
Work-related event(s)	347 (24%)	79 (35%)	268 (22%)	
Only non-coal work related event(s)	300 (21%)	32 (14%)	268 (22%)	NA
Any coal work related event(s)	47 (3%)	47 (21%)	--	NA
Source, Experienced serious Danger or serious Injury				
War zone/war casualties	161 (11%)	23 (10%)	138 (11%)	0.55
Serious danger	132 (9%)	19 (8%)	113 (9%)	0.62
Serious injury	30 (2%)	7 (3%)	23 (2%)	0.31
Automobile/Work/Other Accident	395 (28%)	80 (35%)	315 (26%)	0.01
Serious danger	295 (21%)	66 (29%)	229 (19%)	0.001
Serious injury	174 (12%)	43 (19%)	131 (11%)	0.001
Work-related (any)	150 (11%)	48 (21%)	102 (8%)	< 0.001
Coal-related		27 (12%)		
Major natural/technological disaster	299 (21%)	57 (25%)	242 (20%)	0.09
Serious danger	186 (13%)	34 (15%)	152 (13%)	0.34
Serious injury	23 (2%)	3 (1%)	20 (2%)	0.99
Work-related (any)	110 (8%)	24 (11%)	86 (7%)	0.08
Coal-related		9 (4%)		
Other traumatic situation	301 (21%)	57 (25%)	244 (20%)	0.10
Serious injury	106 (7%)	24 (11%)	82 (7%)	0.05
Work-related (any)	113 (8%)	15 (7%)	98 (8%)	0.43
Coal-related		6 (3%)		
Witnessed serious accident	473 (33%)	85 (37%)	388 (32%)	0.13
Work-related (any)	239 (17%)	60 (26%)	179 (15%)	< 0.001
Coal-related		39 (17%)		NA
Childhood physical abuse	230 (16%)	34 (15%)	196 (16%)	0.61
Sexual trauma, any age	112 (8%)	12 (5%)	100 (8%)	0.12

Source, perceived seriousness of danger and injury based on Brief Trauma Questionnaire responses.

Differences between coal mining and non-coal mining employment tested by Chi square.

NA = Chi square test not applicable.

Table 3 provides the results of multivariable logistic regression modeling of coal mining risk for depression, anxiety, and PTSD, taking into account prior trauma, age, smoking status, obesity, and educational level. Coal mining was associated with 60% increased odds of depression (OR 1.6; 95% CI 1.1 to 2.4) and 73% increased odds of anxiety (OR 1.7; 95% CI 1.1 to 2.7). In contrast, in analysis limited to those with any traumatic event (*n* = 711), coal mining was not associated with increased risk of PTSD in either the unadjusted model or the model adjusting for age, smoking status, obesity, and educational level (OR 0.96 [95% CI 0.6 to 1.5] and OR 0.80 [95% CI 0.5, to 1.3], respectively).

**Table 3:** Association of Coal Mining Exposure and Trauma with Depression, anxiety, and PTSD

	Sample	Frequency n (%)	Unadjusted models	Adjusted models
Depression (PHQ8 ≥ 10)				
All respondents	1428	217 (15%)		
Any coal mining occupation			OR (95% CI)	
No	1201	164 (14%)	Referent	Referent
Yes	227	53 (23%)	1.93 (1.4, 2.7)	1.62 (1.1, 2.4)
Anxiety (GAD7 ≥ 10)				
All respondents	1428	139 (10%)		
Any coal mining occupation			OR (95% CI)	
No	1201	103 (9%)	Referent	Referent
Yes	227	36 (16%)	2.01 (1.3, 3.0)	1.73 (1.1, 2.7)
Post-traumatic Stress (PTSD screener ≥ 3)				
Respondents reporting trauma	711	184 (26%)		
Any coal mining occupation			OR (95% CI)	
No	584	152 (26%)	Referent	Referent
Yes	127	32 (25%)	0.96 (0.6, 1.5)	0.80 (0.5, 1.3)

PHQ-8 has a range of 0–24 points. GAD-7 has a range of 0–21 points; PTSD has a range of 0–5 points.

OR = Odds ratio Odds ratios from logistic regression models.

Adjusted logistic regression models for depression and anxiety control for trauma (work-related or non-work related), age, smoking status, obesity, and educational level (see Table 1). Models for PTSD are limited to those reporting trauma; the adjusted model includes age, smoking status, and educational level.

Further analysis of depression, anxiety, and PTSD risk by source of trauma is presented in Table 4. Non-work-related trauma was not statistically associated with depression, whereas work-related trauma from non-coal and from coal events were associated with similar three-fold elevated odds of depression (OR 3.2 [95% CI 2.2 to 4.5] and OR 3.5 [95% CI 1.8 to 6.8], respectively). In contrast, non-work trauma was associated with increased odds of anxiety (OR 1.6; 95% CI 1.0 to 2.6); non-coal trauma was associated with increased odds of anxiety similar to that for depression (OR 3.2; 95% CI 2.0 to, 5.1); while the odds of anxiety associated with coal trauma were increased six-fold (OR 6.0; 95% CI 2.9 to 12.4). For the analysis of PTSD, limited to those with any trauma (*n* = 711) and those with non-work-related trauma as the referent, the odds of PTSD were more than doubled but were similar for non-coal or coal-related work events.

**Table 4:** Association of Source of Trauma Events with Depression, anxiety, and PTSD

	Sample	Frequency n (%)	Unadjusted models	Adjusted models
Depression (PHQ8 ≥ 10)				
All respondents	1428	217 (15%)		
Source of Trauma			OR (95% CI)	
No traumatic event	717	71 (10%)	Referent	Referent
Only non-work-related event(s)	364	47 (13%)	1.35 (0.9, 2.0)	1.17 (0.8, 1.8)
Non-coal related event(s)	300	84 (28%)	3.54 (2.5, 5.0)	3.15 (2.2, 4.5)
Any coal-related event(s)	47	15 (32%)	4.27 (2.2, 8.3)	3.47 (1.8, 6.8)
Anxiety (GAD7 ≥ 10)				
All respondents	1428	0 (10%)		
Source of Trauma			OR (95% CI)	
No traumatic event	717	39 (5%)	Referent	Referent
Only non-work-related event(s)	364	35 (10%)	1.85 (1.2, 3.0)	1.62 (1.0, 2.6)
Non-coal related event(s)	300	51 (17%)	3.56 (2.3, 5.5)	3.23 (2.0, 5.1)
Any coal-related event(s)	47	14 (30%)	7.38 (3.7, 14.9)	6.03 (2.9, 12.4)
Post-traumatic Stress (PTSD screener ≥ 3)				
Respondents reporting trauma	711	184 (26%)		
Source of Trauma			OR (95% CI)	
Only non-work-related event(s)	364	69 (19%)	Referent	Referent
Non-coal related event(s)	300	98 (33%)	2.07 (1.5, 3.0)	2.15 (1.5, 3.1)
Any coal-related event(s)	47	17 (36%)	2.42 (1.3, 4.6)	2.41 (1.2, 4.7)

Odds ratios from logistic regression models.

Adjusted models control for age, education, smoking, and obesity.

Of the 56 participants who reported a diagnosis of CWP, 28 (50%) reported coal trauma, whereas 19 (11%) of the remaining 171 coal workers reported coal trauma (*p* < 0.001). As shown in Table 4, of the 47 participants with coal trauma, 14 (30%) met the definition of anxiety. Of these 14, 9 (64%) also reported CWP. Excluding all 56 participants with CWP from the analysis, however, did not attenuate the association between coal trauma and anxiety (OR 5.9; 95% CI 1.9 to 18.0).

## Discussion

In this Appalachian region, population-based study, we found that coal miners experience more prevalent work-related trauma than other persons with non-coal employment histories and, associated with that, carry an increased burden of depression and anxiety. For depression, the odds were similar for coal or non-coal work trauma, while for anxiety, trauma from coal work was associated with six-fold increased odds of this adverse outcome. Post-traumatic stress disorder was associated with work trauma as opposed to other sources, but the odds were similar for coal and non-coal work-related events.

These findings support and expand upon the observation of Harris and colleagues of increased risk of morbidity from depression, anxiety, and PTSD among coal miners [4]. Our study cohort was population-based rather than drawn from a clinical care setting as in the Harris study in which only patients seeking care in a coal workers’ health center were interviewed without others from the same region. The inclusion of others from the same region reduces the likelihood such persons had marker health conditions and that they had a wide range of employment histories, reducing that as source of selection bias in the sample. The limited and conflicting biomedical literature, none from the United States except for the study by Harris and colleagues, further underscores the importance of studying such morbidity among miners in the context of other working persons. One small study observed a low prevalence of symptoms of depression and anxiety among current Brazilian coal miners [14]. In contrast, a larger survey limited to active coal miners in China reported a depressive and anxiety prevalence of 19.9 and 49.7%, respectively [15]. Importantly, neither of these studies included referent population data so that the estimates could be compared to other occupational groups. Increased symptoms of depression and anxiety were reported in a study of silicosis in Turkey and increased depression incidence among those with pneumoconiosis in China; neither included a disease-free referent group [16, 17]. Further, symptoms of depression and anxiety also have been reported in an asbestos-exposed cohort in France [18]. A study from Australia reported increased depression and anxiety among workers in a gold mine [19]. Post-traumatic stress disorder in relation to coal mining has received even less attention than depression or anxiety, although this topic has been addressed in a single publication from China, now more than a decade old [20]. There have been several additional studies of psychological stress in miners from Australia using the Kessler Psychological Distress Scale, which subsumes the constructs depression and anxiety, but does not report them separately [21–23]. 

Over the last four decades, the number of persons employed in coal mining in the United States has declined by approximately 75%, although more than one half of those still working in the industry are located in the region of Applachia [24, 25]. Although data on former miners are limited, the population of those who have retired but are still living is likely to be considerable. We surveyed selected counties in multiple states in Appalachia and there are no county-specific population estimates of ever-employment by occupation that would allow proportional comparisons. The population-based random-digit dial telephone survey methodology that we employed in this study is similar to the approach that we have used previously in the study of rheumatologic disease risk among coal miners in Appalachia [7, 8] and hard rock miners in the western United States [26]. This study design allows comparison to other employed persons in the same region who do not have mining employment histories and does not select for persons with morbidity. Surveying in this manner, however, limits exposure classification to self-reported occupational histories and symptoms. Using validated batteries to assess and classify depression, anxiety, and PTSD, is a standard approach to the study of these conditions but has the limitation of potentially greater misclassification than a focused clinical examination. This survey method also allows for selection bias theoretically to be present were those with or without depression, anxiety, or PTSD more likely to participate in a way systematically related to coal mining. Respondents, however, were not informed as part of recruitment that this study was about coal mining. We have no reason to believe that selection bias substantively impacted our findings. Our regional survey limits generalizability to coal miners elsewhere in the United States or in other countries around the globe. Even though we included data from more than 1400 interviews, persons with coal mining histories comprised only just over 200 participants. Our study protocol, by limiting recruitment to males over age 50, few of whom were currently mining, also is an important limitation to generalizability. For these reasons our findings may not be relevant to younger male current workers, nor can we make inferences about work trauma and depression, anxiety, or PTSD among women. Our multivariable modelling is robust but other factors may be associated with unaccounted variability. We did not include reported income as a covariate, not only because it was missing for a substantial minority of participants, but also because of the complex interrelationships among active employment, retirement, disability, income insecurity, and adverse psychological status that are important and clearly merit additional study [27]. In addition, low income in persons in this age range is often the result of early retirement or disability rather than a cause of the problem being studied, in this case depression and anxiety associated with coal mining. In such situations, it is preferable to substitute education for income as a measure of disadvantage since for most individuals, education levels do not change after early adulthood [28]. For that reason, we included education in the multivariable analyses we performed.

In summary, coal mining, well recognized as a risky occupation for work-related physical injury and CWP, is also associated with increased odds of depression, anxiety, and PTSD. Interventions intended to promote coal mining safety and prevent lung disease also should take mental health morbidity into account. More broadly, such morbidity across a range of occupations may warrant focused attention.

## Data Availability

The study questionnaire is available upon request.

## References

[1] Schein J, Houle C, Annette Urganus A. Prevalence of post-traumatic stress disorder in the United States: a systematic literature review. Cur Med Res Opin. 2021;37:2151–61. 10.1080/03007995.2021.1978417 34498953

[2] Goodwin RD, Dierker LC, Wu M, Galea S, Hoven CW, Weinberger AH. Trends in U.S. depression prevalence from 2015 to 2020: the widening treatment gap. Am J Prev Med. 2022;63:P726–733. 10.1016/j.amepre.2022.05.014 PMC9483000 36272761

[3] Goodwin RD, Weinberger AH, Kim JH, Wu M, Galea S. Trends in anxiety among adults in the United States, 2008–2018: Rapid increases among young adults. J Psychiat Res. 2020;130:441–6. 32905958 10.1016/j.jpsychires.2020.08.014 PMC7441973

[4] Harris D, McMurry T, Caughron A, Willis J, Blackburn JC, Brizendine C, Tomann M. Characterization of mental illness among US coal miners. JAMA Netw Open. 2021;4(5):e2111110. 34032856 10.1001/jamanetworkopen.2021.11110 PMC8150676

[5] José M, Pizarro J, Aguayo Fuenzalida F. Mental health in mine workers: a literature review. Ind Health. 2021;59:343–70. 34588377 10.2486/indhealth.2020-0178 PMC8655752

[6] National Institute for Occupational Safety and Health (Centers for Disease Control). Coal workers’ pneumoconiosis: top 50 counties with highest age-adjusted death rates (per million population), U.S. residents age 15 and over, 2000–2009 https://wwwn.cdc.gov/eworld/Data/Coal_Workers_Pneumoconiosis_Top_50_counties_with_highest_age-adjusted_death_rates_per_million_population_US_residents_age_15_and_over_20002009/767 (accessed 13 October 2018).

[7] Schmajuk G, Trupin L, Yelin E, Blanc PD. Prevalence of arthritis and rheumatoid arthritis in coal mining counties of the U.S. Arthritis Care Res (Hoboken). 2019;71:1209–15. 30875457 10.1002/acr.23874 PMC6717008

[8] Schmajuk G, Trupin L, Yelin EH, Blanc PD. Dusty trades and associated rheumatoid arthritis in a population-based study in the coal mining counties of Appalachia. Occup Environ Med. 2022;79:308–14. 34987082 10.1136/oemed-2021-107899

[9] Bovin MJ, Kimerling R, Weathers FW. Diagnostic accuracy and acceptability of the primary care posttraumatic stress disorder screen for the Diagnostic and Statistical Manual of Mental Disorders (Fifth Edition) among US veterans. JAMA Netw Open, 2021; 4(2), e203673313. 10.1001/jamanetworkopen.2020.36733 PMC7862990 33538826

[10] Schurr P, Vielhauer M, Weathers F, Findler M. March. The Brief Trauma Questionnaire (BTQ) https://www.ptsd.va.gov/professional/assessment/documents/BTQ.pdf (accessed 3 2024).

[11] Kroenke K, Strine TW, Spitzer RL, Williams JBW, Mokdad AH. The PHQ-8 as a measure of current depression in the general population. J Affect Disorders 2009; 114: 163‐173.10. 10.1016/j.jad.2008.06.026 18752852

[12] Spitzer RL, Kroenke K, Williams JBW, Löwe B. A brief measure for assessing generalized anxiety disorder: the GAD-7. Arch Intern Med. 2006;11:1661092–97. 10.1001/archinte.166.10.1092 16717171

[13] Toussainta A, Hüsinga P, Gumza A et al. Sensitivity to change and minimal clinically important difference of the 7-item generalized anxiety disorder questionnaire (GAD‐7). J Affect Disord 2020; 265: 395‐401.12. 10.1016/j.jad.2020.01.032 32090765

[14] Joaquim AC, Lopes M, Stangherlin L. Et. Al. Mental health in underground coal miners. Arch Environ Occup Health. 2018;73:334–43. 29279016 10.1080/19338244.2017.1411329

[15] Liu L, Wen F, Xu X, Wang L. Effective resources for improving mental health among Chinese underground coal miners: perceived organizational support and psychological capital. J Occup Health. 2015;57:58–68. 25410268 10.1539/joh.14-0082-OA

[16] Yildiz T, Eşsizoğlu A, Onal S. Et. Al. Quality of life, depression and anxiety in young male patients with silicosis due to denim sandblasting. Tuberk Toraks. 2011;59:120–5. 21740385 10.5578/tt.1606

[17] Lee HM, Liu DY, Hsu HL. Risk of depression in patients with pneumoconiosis: a population-based retrospective cohort study. J Affect Disord. 2024;352:146–52. 38369263 10.1016/j.jad.2024.02.057

[18] Mounchetrou Njoya I, Paris C, Dinet J. Et. Al. Anxious and depressive symptoms in the French asbestos-related diseases Cohort: risk factors and self-perception of risk. Eur J Public Health. 2017;27:359–66. 27452893 10.1093/eurpub/ckw106

[19] Velander F, Schineanu A, Liang W, Midford R. Digging for gold and coming up blue: a health survey in the mining industry. J Occup Health Saf Aust N Z. 2010;26:389–401.

[20] Wang HH, Zhang ZJ, Tan QR. Psychopathological, biological, and neuroimaging characterization of posttraumatic stress disorder in survivors of a severe coalmining disaster in China. J Psychiatr Res. 2010;44:385–92. 19896142 10.1016/j.jpsychires.2009.10.001

[21] James C, Rahman M, Bezzina A, Kelly B. Factors associated with patterns of psychological distress, alcohol use and social network among Australian mineworkers. Aust N Z J Public Health. 2020;44:390–6. 32865849 10.1111/1753-6405.13037

[22] Considine R, Tynan R, James C, Wiggers J, Lewin T, Inder K, Perkins D, Handley T, Kelly B. The contribution of individual, social and work characteristics to employee mental health in a coal mining industry population. PLoS ONE. 2017;12(1):e0168445. 28045935 10.1371/journal.pone.0168445 PMC5207427

[23] Bowers J, Lo J, Miller P, Mawren D, Jones B. Psychological distress in remote mining and construction workers in Australia. Med J Aust. 2018;208:391–7. 29747563 10.5694/mja17.00950

[24] U.S. Bureau of Labor Statistics, Employees A. Coal Mining [CES1021210001], retrieved from FRED, Federal Reserve Bank of St. Louis; https://fred.stlouisfed.org/series/CES1021210001. Accessed 9 October 2024.

[25] U.S. Energy Information Administration Annual Coal Report. 2022. Table 18. Average Number of Employees by State and Mine Type, 2022 and 2021 https://www.eia.gov/coal/annual/pdf/table18.pdf Accessed 9 October 2024.

[26] Blanc PD, Trupin L, Yelin EH, Schmajuk G. Assessment of risk of rheumatoid arthritis among underground hard rock and other mining industry workers in Colorado, New Mexico, and Utah. JAMA Netw Open. 2022;5:e2236738. 36251293 10.1001/jamanetworkopen.2022.36738 PMC9577677

[27] Yuan B. How the interplay of late retirement, health care, economic insecurity, and electronic social contact affects the mental health amongst older workers? Stress Health. 2024;40(2):e3309. 37621258 10.1002/smi.3309

[28] Haveman R, Wolfe B, Buron L, Hill SC. The loss of earnings capability from disability/health limitations: toward a new social indicator. Rev Income Wealth. 1995;41:289–308.

